# Testlet-Based Multidimensional Adaptive Testing

**DOI:** 10.3389/fpsyg.2016.01758

**Published:** 2016-11-18

**Authors:** Andreas Frey, Nicki-Nils Seitz, Steffen Brandt

**Affiliations:** ^1^Department of Research Methods in Education, Institute of Educational Science, Friedrich Schiller University JenaJena, Germany; ^2^Faculty of Education, Centre for Educational Measurement, University of OsloOslo, Norway; ^3^Art of ReductionAltenholz, Germany

**Keywords:** computerized adaptive testing, item response theory, multidimensional IRT models, testlets, large-scale assessment

## Abstract

Multidimensional adaptive testing (MAT) is a highly efficient method for the simultaneous measurement of several latent traits. Currently, no psychometrically sound approach is available for the use of MAT in testlet-based tests. Testlets are sets of items sharing a common stimulus such as a graph or a text. They are frequently used in large operational testing programs like TOEFL, PISA, PIRLS, or NAEP. To make MAT accessible for such testing programs, we present a novel combination of MAT with a multidimensional generalization of the random effects testlet model (MAT-MTIRT). MAT-MTIRT compared to non-adaptive testing is examined for several combinations of testlet effect variances (0.0, 0.5, 1.0, and 1.5) and testlet sizes (3, 6, and 9 items) with a simulation study considering three ability dimensions with simple loading structure. MAT-MTIRT outperformed non-adaptive testing regarding the measurement precision of the ability estimates. Further, the measurement precision decreased when testlet effect variances and testlet sizes increased. The suggested combination of the MTIRT model therefore provides a solution to the substantial problems of testlet-based tests while keeping the length of the test within an acceptable range.

## Introduction

Multidimensional adaptive testing (MAT) is a highly efficient method for the simultaneous measurement of several latent traits (e.g., Frey and Seitz, 2009; Segall, 2010). MAT has two major benefits. First, theoretical assumptions about multidimensional structures of the constructs of interest can be directly incorporated into the measurement instrument by using multidimensional item response theory (MIRT; e.g., Reckase, 2009) models as measurement models. Second, the measurement efficiency of MAT is substantially higher compared to unidimensional adaptive testing (UCAT) or non-adaptive sequential testing if correlations between the measured constructs are considered in the item selection process and for ability estimation (Segall, 1996; Wang and Chen, 2004; Frey and Seitz, 2009; Frey et al., 2013). Even though MAT performed very well in the simulation studies mentioned, empirical applications in large testing programs are still missing.

The current lack of operational MAT applications might be due to the relative inflexibility of the “pure” MAT algorithms which were formulated at the onset of MAT-related research in the 1990s (Luecht, 1996; Segall, 1996; van der Linden, 1999). Reflecting this, more current research activities are focusing on extensions which increase the flexibility of MAT. Makransky et al. (2013), for example, showed how MAT can be used with polytomous items. Other papers have focused on employing complex model structures in MAT (Huang et al., 2012; Wang, 2014; Wang et al., 2014; Mikolajetz and Frey, in press), on MAT with exposure control (Lee et al., 2008; Diao et al., 2009; Finkelman et al., 2009), on the simultaneous consideration of multiple restrictions in MAT (Veldkamp and van der Linden, 2002; Su, 2016; Born and Frey, in press), on online calibration (Chen and Wang, 2016) and on comparing different item selection methods for composite scores and sub scores in general (Yao, 2012), with respect to different test termination criteria (Yao, 2013), and different exposure control and content managing methods (Yao, 2014).

At present, the application of MAT may also be hindered by the fact that it is formulated at the level of items while procedures to adequately process item pools consisting of testlets are missing. Testlets are sets of items sharing the same stimulus such as a graph, a picture, a reading passage, or other context elements. They are used, for example, in nearly all major large-scale assessments of student achievement such as PISA, PIRLS, or NAEP.

Optimally, a new approach for using testlets in MAT should provide a solution to the frequently reported problem of local item dependence (LID) between the items of the same testlet, which is typically not yet addressed in operational large-scale assessments. LID is present if non-zero inter-correlations between items remain after the level of the latent trait or latent traits measured by the items has been controlled for. In several studies, LID between the items of a testlet has been observed (e.g., Monseur et al., 2011), leading to a substantial overestimation of test information and scale reliability, and corresponding underestimations of the standard errors of the latent trait estimates (Sireci et al., 1991; Yen, 1993; Wang and Wilson, 2005a; Marais and Andrich, 2008). These effects occur because the systematic variance introduced by LID is in part attributed to the ability variable. However, since this portion of variance is just caused by the way the instrument is composed and not by the ability level of the test person, this is not appropriate. Rather, it has to be regarded as a severe problem, since it means that tests of significance (e.g., for comparisons of skill means between girls and boys or between countries) are based on standard errors that are too small and therefore produce too many significant results. Additionally, LID can lead to errors in scaling and equating (Lee et al., 2001; Li et al., 2005) and biased item discrimination parameter estimates (Ackerman, 1987). Regarding MIRT models, Brandt (2012) reported that covariance estimates are also systematically biased in the presence of LID. It is important to note that even though substantial amounts of LID seem to be present in testlet-based test data and that LID has unwanted effects on the parameter estimates of interest, this issue has not yet been addressed by appropriate psychometric models in large-scale testing programs; this seems very problematic.

Even though addressing LID with appropriate psychometric models would be necessary in order to avoid the above mentioned problems, the high complexity of such models would either necessitate prolonging testing sessions, if comparable standard errors of the statistics of interest should be obtained, or reducing the amount or grade of differentiation of the measured content. Both would be problematic for most large-scale assessment programs. In any case, LID is a serious problem that has the potential to spuriously boost observed measurement precision and reliability estimates and to jeopardize the inferences derived from large-scale assessment results. Thus, LID is an issue psychometrics has to face and solve in order to provide unbiased and efficient estimates as prerequisites of valid test score interpretations. As mentioned above, MAT has shown to have very high measurement efficiency and, furthermore, provides a possibility to use an appropriate psychometric model for testlet-based tests. With this flexibility, MAT avoids unwanted effects on parameter estimates of interest, without increasing the length of testing sessions or limiting the measured content.

This study presents and evaluates a new method that combines the merits of a complex psychometric model with testlet effects with the above mentioned very high measurement efficiency of MAT (compared to non-adaptive sequential testing and UCAT). Thereby, (a) MAT's applicability is expanded to the large group of testlet-based tests, and (b) an adequate solution to the problem of how to handle LID between the items of a testlet is demonstrated.

The rest of the text is organized as follows: First, a MIRT model parameterizing LID between the items of the same testlet is introduced. Next, a possibility for how to combine this model with MAT is described. Based on this, the research questions for the simulation study carried out to evaluate the suggested combination are stated. Then, the methods and results of the simulation study are presented. Finally, the results and their implications for practical applications of testlet-based MAT are discussed.

## Multidimensional testlet model

Several psychometric models have been proposed to account for LID between the items of a testlet. One of the first was presented by Rosenbaum (1988), who suggested treating each testlet as a polytomous item. A testlet entailing four items, for example, is considered as a polytomous item with five response categories and scaled with a polytomous IRT model like the partial credit model (Masters, 1982) or the rating scale model (Andrich, 1978). Using polytomous IRT models to account for LID between items embedded in testlets is generally appropriate if the underlying assumption of conditional independence between different testlets holds. However, this approach has the disadvantage that by treating a testlet as a single item with several response categories, only the sum of the correct responses to the items assembled in a testlet is modeled; more differentiated information of the individual responses to the single items is not considered. This results in an unnecessary loss of information and other problems, like, for example, difficulties to build up proficiency levels (for a more detailed discussion, see Wainer et al., 2007).

An alternative model which retains the information of single items and parameterizes effects caused by LID is the testlet IRT (TIRT) model with random effects. It was introduced by Bradlow et al. (1999) and generalized by Wainer et al. (2000). The latter is basically the 3PL model supplemented by a person-specific testlet effect. If each item *i* of a test is nested in exactly one testlet *d* of *D* mutually exclusive testlets, the probability of a correct answer *u* of person *j* = 1, …, *N* with ability θ_*j*_ to an item is given in this model by

(1) $$\begin{array}{l} P\left(U_{ij}=1|\theta_{j},a_{i},b_{i},c_{i},\gamma_{jd\left(i\right)}\right)\;=\\ c_{i}+\left(1-c_{i}\right)\frac{\text{exp}\left(a_{i}\left(\theta_{j}-b_{i}-\gamma_{jd\left(i\right)}\right)\right)}{1+\text{exp}\left(a_{i}\left(\theta_{j}-b_{i}-\gamma_{jd\left(i\right)}\right)\right)}\;. \end{array}$$

The difficulty, discrimination, and pseudo-guessing parameters of item *i* are denoted with *b*_*i*_, *a*_*i*_, and *c*_*i*_, respectively. Additionally, the so-called testlet effects, γ_*jd*(*i*)_, are introduced to model LID between the items of testlet *d*. They can be regarded as testlet-specific random nuisance factors, modeling systematic variance caused by LID without affecting the mean of θ_*j*_. To achieve this and in order for the model to be identified, θ_*j*_ and γ_*jd*(*i*)_ are assumed to be uncorrelated and normally distributed with the mean and the variance of the θ_*j*_ distribution and the mean of the γ_*jd*(*i*)_ distribution being fixed (e.g., means set to 0 and variance set to 1). Imposing these restrictions is sufficient for the model to be identified and makes unequivocal interpretations of θ_*j*_ and γ_*d*(*i*)_ possible. In practice, the estimated variances $\sigma_{\gamma_{d\left(i\right)}}^{2}$ of the testlet effects are especially informative, because they can be seen as indicators of the degree of LID within the corresponding testlet *d*.

For the estimation of the model parameters, Wainer et al. (2000) proposed embedding the model given by Equation (1) in a larger hierarchical Bayesian framework with priors given by θ_*j*_~*N*(0, 1), $a_{i}\sim N\left(\mu_{a},\sigma_{a}^{2}\right),b_{i}\sim N\left(\mu_{b},\sigma_{b}^{2}\right)$, $\text{log}\left(c_{i}/\left(1-c_{i}\right)\right)\sim N\left(\mu_{c},\sigma_{c}^{2}\right)$, and $\gamma_{jd\left(i\right)}\sim N\left(0,\sigma_{\gamma_{d\left(i\right)}}^{2}\right)$. The means of the distributions are set to μ_*a*_~*N*(0, *V*_*a*_), μ_*b*_~*N*(0, *V*_*b*_), and μ_*c*_~*N*(0, *V*_*c*_), where $V_{a}^{-1}=V_{b}^{-1}=V_{c}^{-1}=0$. For all prior variances, slightly informative hyperpriors were used, given by $\sigma_{z}^{2}\sim \chi_{g_{z}}^{-2}$, an inverse chi-square distributed random variable with *g*_*z*_ degrees of freedom, with *g*_*z*_ set to 0.5. These distributional assumptions are typical for Bayesian analyses of high dimensional IRT models. Further information can be found in Wainer et al. (2007). Unknown parameters are estimated by drawing samples from their marginal posterior distributions using Markov Chain Monte Carlo (MCMC) modeling. Wainer et al. (2000) provide comprehensive simulation results, underlining that the suggested model, in conjunction with the proposed manner of estimation, is very powerful in estimating not only difficulty parameters and abilities but also item discriminations. The mean absolute bias, for example, was about 26% smaller for the model in Equation (1) with MCMC estimation than for a conventional 3PL model estimated with marginal maximum likelihood (MML). Since the item information and reliability are functions of the item discriminations, the model is thus suitable to overcome the problems introduced by LID mentioned above. A successful application of the model in Equation (1) for item selection and ability estimation in a testlet-based UCAT can be found in Keng (2011).

A closely related approach for considering LID between items of a testlet is the *Bi-Factor Full-Information Factor Analysis Model* (Gibbons and Hedeker, 1992) which was developed out of the factor analytic tradition. This model is referred to as *bi-factor model* in the following sections. As the random effects testlet model, the bi-factor model includes a general ability dimension θ and a testlet-specific dimension θ_*d*(*i*)_. In contrast to the random effects testlet model in Equation (1), the item discriminations are allowed to have different values for the general ability dimension and the testlet dimension in the bi-factor model. Formally, it is given by

(2) $$\begin{array}{l} P\left(U_{ij}=1|\theta_{j},\theta_{jd\left(i\right)},a_{i},b_{i},c_{i}\right)\;=\\ c_{i}+\left(1-c_{i}\right)\frac{\text{exp}\left(a_{i1}\theta_{j}-a_{i2}\theta_{jd\left(i\right)}-b_{i}\right)}{1+\text{exp}\left(a_{i1}\theta_{j}-a_{i2}\theta_{jd\left(i\right)}-b_{i}\right)}\;. \end{array}$$

Li et al. (2006) showed that the random effects testlet model is a special case of the bi-factor model. It can be derived from the bi-factor model by fixing the loadings of the items on the testlet dimension proportional to their loadings on the ability dimension, which is equivalent to the second order model; that is, the testlet model and the higher order model can be transferred into each other (see also Rijmen, 2010). Due to its larger flexibility, achieved by the larger number of parameters, the bi-factor model can achieve a better model fit than the random effects testlets model. Nevertheless, the differences between the two models that have been found in simulation studies and real data applications are relatively small (e.g., DeMars, 2006). A drawback of the higher complexity of the bi-factor model lies in a limited applicability in operational testing programs. Furthermore, it tends to overestimate the testlet slopes in cases where no testlet effects are present in the data (DeMars, 2006) while the TIRT model does not. Hence, in the following sections, the TIRT model is further considered. However, because both models are so closely related, similar results can be expected for the bi-factor model.

To provide a testlet model for MAT, we suggest generalizing the model in Equation (1) to the multidimensional case. For this purpose, we replaced the ability parameter θ by the ability vector **θ** = (θ_1_, …, θ_*P*_) entailing the abilities for *P* dimensions, and the discrimination parameter *a*_*i*_ for item *i* by the 1 × *P* discrimination vector **a**_*i*_′. Furthermore, *b*_*i*_ and γ_*jd*(*i*)_ are both multiplied with the *P* × 1 vector **1** filled with ones in order to use these parameters for all measured dimensions. The multidimensional IRT random effect testlet (MTIRT) model is then expressed as

(3) $$\begin{array}{l} P\left(U_{ij}=1|\theta_{j},a_{i},b_{i},c_{i},\gamma_{jd\left(i\right)}\right)=\\ \;c_{i}+\left(1-c_{i}\right)\frac{\exp\left(a_{i}\prime \left(\theta_{j}-b_{i}1-\gamma_{jd\left(i\right)}1\right)\right)}{1+\exp\left(a_{i}\prime \left(\theta_{j}-b_{i}1-\gamma_{jd\left(i\right)}1\right)\right)} \end{array}$$

As for the unidimensional random effects testlet model, the estimations of $\sigma_{\gamma_{d\left(i\right)}}^{2}$ are especially useful because they represent the degree of LID between the items of testlet *d*. All other model parameters can be interpreted in the same way as in a conventional multidimensional 3PL model (M3PL). Some constraints need to be imposed in order for the model to be identified and for unequivocal interpretations of **θ** and $\sigma_{\gamma_{d\left(i\right)}}^{2}$ to be possible. Following the assumptions made for the unidimensional random effects testlet model in Equation (1), for its multidimensional generalization, the γ_*jd*(*i*)_ parameters are assumed to be uncorrelated with each other and with **θ**_*j*_, while mutual correlations between the *P* ability dimensions are allowed. Hence, the γ_*jd*(*i*)_ parameters for the model in Equation (3) can also be regarded as testlet-specific random nuisance factors, modeling systematic variance caused by LID without affecting the means of **θ**_*j*_. Further, the ability dimensions θ_1_, …, θ_*P*_ and the testlet dimensions γ_1(*i*)_, …, *y*_*D*(*i*)_ are assumed to be normally distributed with means fixed (e.g., to 0) and the variances of the distributions for the ability dimensions also fixed (e.g., to values known from a previous study or to 1). As an alternative to fixing the ability variances, at least one *a*-parameter can be fixed per dimension (typically to 1). When estimating the complete model, including multidimensional ability **θ**_*j*_, item parameters **a**_*i*_, *b*_*i*_, and *c*_*i*_, and testlet parameters γ_*jd*(*i*)_, MCMC-estimation in a Bayesian framework is recommended, because the superiority of this kind of estimation compared to MML estimation reported for the unidimensional TIRT model (Wainer et al., 2000) can be transferred to the multidimensional case. As usual, priors should be specified to best fit the circumstances of the respective study. A typical specification would be: **θ**_*j*_~*MVN*(**μ**_**θ**_, **Φ**_**θ**_), **a**_*i*_~*MVN*(**μ**_**a**_, **Φ**_**a**_), $b_{i}\sim N\left(\mu_{b},\sigma_{b}^{2}\right)$, $\text{log}\left(c_{i}/\left(1-c_{i}\right)\right)\sim N\left(\mu_{c},\sigma_{c}^{2}\right)\;$, and $\gamma_{jd\left(i\right)}\sim N\left(0,\sigma_{\gamma_{d\left(i\right)}}^{2}\right)$ where **μ**_**θ**_ is the *P*-dimensional vector containing the means of the ability dimensions all fixed at 0 and **Φ**_**θ**_ the *P* × *P* variance-covariance matrix for the ability dimensions with the variances fixed at 1. Means and variances for the unidimensional parameters *b*_*i*_, *c*_*i*_, and γ_*jd*(*i*)_ are specified as described above for the unidimensional testlet model of Wainer et al. (2000). The mean of the distribution of the item discriminations is set to **μ**_**a**_~*MVN*(**0**, **V**_**a**_) where **0** is the *P*-dimensional zero vector and the variance-covariance matrix ${{\text{V}}_{\text{a}}}^{-1}=0$. For the prior variance of the distribution of the item discriminations, slightly informative multidimensional hyperpriors are used, given by $\Phi_{\text{a}}\;\sim W^{-1}\left({\text{V}}_{\text{a}},n\right)$, the inverse Wishart distribution with *n* degrees of freedom as the multidimensional equivalent for the inverse chi-square distribution and **V**_**a**_ as defined before. Note that the number of additional parameters that need to be estimated for the multidimensional model in Equation (3) is not that much larger (item discriminations and covariances between ability dimensions) than under its unidimensional predecessor. Thus, it is a promising candidate to correspond to the simulation results of the unidimensional version and thus makes it possible to accurately estimate all included item parameters and abilities. This expectation is even stronger if reliable information about some of the model parameters is available. This, for example, is the case in CAT where item parameters are estimated beforehand in a calibration study. In such cases, where item parameters are fixed to the values from the calibration study, standard estimation techniques such as MML estimation with Newton Raphson integration should also provide provisional ability and testlet effect estimations with reasonable accuracy.

Just as with the conventional M3PL without testlet effects, the 2PL version of the MTIRT model results if *c* is set to 0. This model can in turn be regarded as a Rasch version of the MTIRT model if the components of **a**_*i*_′ are allowed to have the values 1 and 0 only, indicating whether an item loads on a dimension or not.

**Figure 1:**
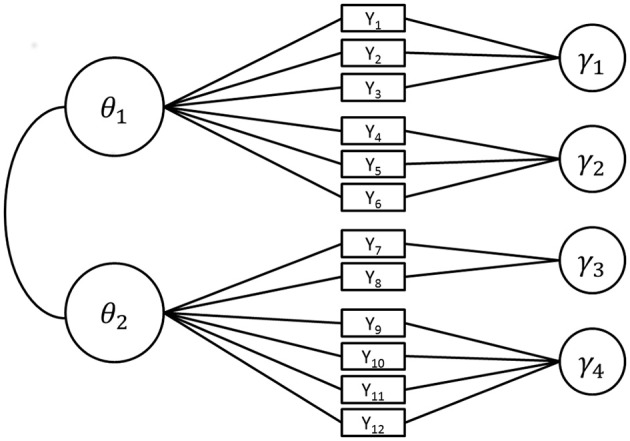
**Path diagram of the structure of a testlet model for 12 items (Y_**1**_ to Y_**12**_) each loading on one of two ability variables θ_**1**_ and θ_**2**_**. Every item belongs to exactly one of four testlets γ_1_ to γ_4_. For identifiability, the means of all latent variables are set to 0 and the variances of θ_1_ and θ_2_ to 1.

Figure 1 shows the structure of the model in Equation (3) for a hypothetical test with two ability dimensions with distinct sets of items loading on each dimension (between item multidimensionality) with a total of 12 items nested in four testlets.

## Combination of the multidimensional random effects testlet model with multidimensional adaptive testing

Several methods have been proposed for item selection in MAT such as maximizing the determinant of the Fisher information matrix (Segall, 1996), minimizing the trace of the inverse Fisher information matrix (van der Linden, 1999), maximizing the posterior expected Kullback-Leiber information (Veldkamp and van der Linden, 2002), maximizing a simplified Kullback-Leibler information index (Wang et al., 2011), and maximizing the mutual information between the current posterior distribution of **θ** and the response distribution on the candidate item (Mulder and van der Linden, 2010). One of the most studied item selection methods for MAT is maximizing the determinant of the Fisher information matrix as proposed in terms of a Bayesian approach by Segall (1996) which is also referred to as the *D*-optimality criterion. This item selection method constantly ranged within the best performing methods with regard to typical evaluation criteria as (conditional) accuracy and precision of ability estimates when compared to other item selection methods (e.g., Veldkamp and van der Linden, 2002; Mulder and van der Linden, 2009; Wang and Chang, 2011; Wang et al., 2011; Yao, 2012, 2013, 2014). It proved to be a robust method over a broad range of MAT specifications even though in some studies other item selection methods performed slightly better (e.g., mutual information in Wang and Chang, 2011). However, since Segall's item selection method was performing well in the mentioned studies and with many MAT configurations, no study pin-pointed problems associated with the method even when complex models were used, and the fact that it was successfully applied by Wang (2014) in MAT with the higher order IRT model (e.g., de la Torre and Song, 2009) which is closely related to the testlet model, it was adopted as the item selection method of choice for the present study. Since the aim of our study is to specify an applicable solution for testlet-based MAT for the first time, we focus on Segall's item selection method and do not compare different item selections methods. Future studies might compare different methods, even though only small differences between the best performing methods (*D*-optimality, mutual information, Kullback-Leibler index) are to be expected based on the available results.

One important aspect of the method proposed by Segall (1996) lies in using the variance-covariance matrix **Φ** of the measured latent traits as prior information. The matrix **Φ** is of size *P* × *P* and has the following general structure:

(4) $$\begin{array}{l} \Phi =\left(\begin{array}{cccc} \sigma_{\theta_{1}}^{2} & \sigma_{\theta_{1},\theta_{2}} & \ldots & \sigma_{\theta_{1},\theta_{P}}\\ \sigma_{\theta_{2},\theta_{1}} & \sigma_{\theta_{2}}^{2} & \ldots & \sigma_{\theta_{2},\theta_{P}}\\ \vdots & \vdots & \ddots & \vdots \\ \sigma_{\theta_{P},\theta_{1}} & \sigma_{\theta_{P},\theta_{2}} & \ldots & \sigma_{\theta_{P}}^{2} \end{array}\right) \end{array}$$

**Φ** is used for the estimation of the provisional abilities within the test and the final abilities at the end of the test as well as for the selection of items from the pool. For the estimation of the latent abilities, Segall (1996) proposed using the multidimensional Bayes modal estimator with Fisher scoring in combination with **Φ**. Regarding item selection, he suggested selecting the one item from all eligible items for presentation which maximizes the quantity

(5) $$\begin{array}{l} |{\text{W}}_{t+i^{*}}|=|\Phi^{-1}+\text{I }\left(\theta ,{\hat{\theta }}_{j}\right)+\text{I }\left(\theta ,u_{i^{*}}\right)|. \end{array}$$

The matrix ${\text{W}}_{t+i^{*}}$ has the size *P* × *P* and is the sum of three matrices. The first, **Φ**^−1^, is the inverse of the variance-covariance matrix as defined in Equation (4). The second, $\text{I }\left(\theta ,{\hat{\theta }}_{j}\right)$, is the information matrix for **θ** of the previously *t* administered items and the estimated thetas (${\hat{\theta }}_{j}$). According to Segall (1996), the elements of this *P* × *P* matrix with *r* rows and *s* columns, for the M3PL are given by the negative expectation of the second partial derivative of the log likelihood:

(6) $$\begin{array}{l} {\text{I}}_{rs}\left(\theta ,\hat{\theta }\right)=-\text{E}\left[\frac{\partial^{2}\ln\;L}{\partial \theta_{r}\partial \theta_{s}}\right]. \end{array}$$

The diagonal elements of this matrix take the form

(7) $$\begin{array}{l} {\text{I}}_{rr}\left(\theta ,\hat{\theta }\right)=-\overset{t}{\underset{i=1}{\sum }}\frac{a_{ri}^{2}Q_{i}\left(\theta \right)\left(P_{i}\left(\theta \right)-c_{i}\right)\left(c_{i}P_{i}\left(\theta \right)-P_{i}^{2}\left(\theta \right)\right)}{P_{i}^{2}\left(\theta \right){\left(1-c_{i}\right)}^{2}}\\ =\overset{t}{\underset{i=1}{\sum }}\frac{{\left(\frac{\partial P_{i}\left(\theta \right)}{\partial \theta_{r}}\right)}^{2}}{P_{i}\left(\theta \right)Q_{i}\left(\theta \right)}, \end{array}$$

where *Q*_*i*_ = 1 − *P*_*i*_.

The off-diagonal elements are given by

(8) $$\begin{array}{l} {\text{I}}_{rs}\left(\theta ,\hat{\theta }\right)=-\overset{t}{\underset{i=1}{\sum }}\frac{a_{re}a_{si}Q_{i}\left(\theta \right)\left(P_{i}\left(\theta \right)-c_{i}\right)\left(c_{i}P_{i}\left(\theta \right)-P_{i}^{2}\left(\theta \right)\right)}{P_{i}^{2}\left(\theta \right){\left(1-c_{i}\right)}^{2}}\\ =\overset{t}{\underset{i=1}{\sum }}\frac{\frac{\partial P_{i}\left(\theta \right)}{\partial \theta_{r}}\times \frac{\partial P_{i}\left(\theta \right)}{\partial \theta_{s}}}{P_{i}\left(\theta \right)Q_{i}\left(\theta \right)}. \end{array}$$

The third summand, $\text{I }\left(\theta ,u_{i^{*}}\right)$, is the information matrix for **θ** of a response $u_{i^{*}}$ to item i^*^. It has the same form as specified by Equations (6)–(8), with the difference that it represents only one item *i* and is not summed across the *t* presented items. In the course of the test, item i^*^ is selected from the item pool, which results in the largest determinant of the matrix ${\text{W}}_{t+i^{*}}$. This item provides the largest decrement in the volume of the credibility ellipsoid around the current estimation of the latent ability vector ${\hat{\theta }}_{j}$. Thus, after the test calibration, besides the item parameters, the variance-covariance matrix **Φ** is considered to be known for operational test use. Since the elements of **Φ** are group statistics, they are not necessarily correct for any tested individual. But, the importance of **Φ** in relation to the information stemming from the given responses diminishes quickly when the test moves on. For a reasonable test, even mis-specified **Φ** matrices have a negligible impact on the ability estimates (Yoo and Hambleton, 2013). However, for short tests and/or if important decisions need to be made on an individual level, **Φ** does not need to be fixed. In this case, **Φ**^−1^ can be dropped from Equation (5).

Since it is not feasible for a testlet-based test to present single items out of a testlet, Equation (5) cannot be directly combined with the model in Equation (3) to build a testlet-based MAT. Two modifications are needed in order to apply the rationale behind Equations (4) and (5) for tests which are composed of testlets.

### Modification 1: testlet information instead of item information

Firstly, an information measure for complete testlets instead of single items needs to be at hand for selecting complete testlets for presentation instead of single items. Several procedures have been used to calculate such a measure. A commonly applied procedure (e.g., Keng, 2011 or Murphy et al., 2010 for the unidimensional case) to calculate the testlet information is to sum up the item information of all items *i* = 1, …, *I* of a candidate testlet d^*^:

(9) $$\begin{array}{l} \text{I }\left(\theta ,{\text{u}}_{d^{*}}\right)=\overset{I}{\underset{i=1}{\sum }}\text{I }\left(\theta ,u_{d^{*}\left(i\right)}\right). \end{array}$$

Note that, the sum of the item information is only a good choice if all testlets of the test are of the same size. If the testlets differ in the number of items, testlets entailing more items will obviously be favored compared to smaller testlets. In this case, the mean item information of the items of the same testlet is an alternative to using the sum.

The estimation of the testlet information within a MAT process is straightforward: As the testlet effects are defined at the level of individuals, they are not known for the candidate testlets because they have not yet been answered by the participant. Therefore, $\gamma_{jd^{*}\left(i\right)}$ is set to the expectation of 0 for all items of the remaining candidate testlets (Murphy et al., 2010). Hence, the testlet information is not affected by the testlet effect variance for candidate testlets. As a result, the MTIRT model boils down to the MIRT model in the case of information estimation for candidate testlets (detailed information about the elements of $\text{I }\left(\theta ,{\text{u}}_{d^{*}}\right)$ are described below in the part regarding the second modification). This aspect is in fact a strength of the proposed method: Even though it offers an adequate treatment of LID between the items of a testlet (by considering LID for ability estimation), within the course of the test, the data required for the selection of testlets is the same as for the established MAT with a conventional MIRT model. Furthermore, the estimation of provisional ability and testlet parameters during the test can be carried out with MML, providing a solution that is fast enough for operational testing.

### Modification 2: expansion of matrices

Secondly, the variance covariance matrix **Φ** and the information matrices needed for item selection have to be expanded successively as the test moves on in order to apply the MTIRT model in MAT. An additional testlet effect γ_*jd*(*i*)_–which is technically an additional dimension–has to be included in the matrix **Φ** after the items of the corresponding testlet have been answered. Since one more testlet effect can be estimated after the completion of every testlet, the matrix **Φ** has to be expanded after each testlet by one row and one column. After *v* testlets have been presented, the expanded matrix **Φ**_*v*_ is of size (*P* + *v*) × (*P* + *v*) and inherits the following structure:

(10) $$\begin{array}{l} \Phi_{v}=\left(\begin{array}{cccccccc} \sigma_{\theta_{1}}^{2} & \sigma_{\theta_{1},\theta_{2}} & \ldots & \sigma_{\theta_{1},\theta_{P}} & 0 & 0 & \cdots & 0\\ \sigma_{\theta_{2},\theta_{1}} & \sigma_{\theta_{2}}^{2} & \ldots & \sigma_{\theta_{2},\theta_{P}} & 0 & 0 & \cdots & 0\\ \vdots & \vdots & \ddots & \vdots & \vdots & \vdots & \ddots & \vdots \\ \sigma_{\theta_{P},\theta_{1}} & \sigma_{\theta_{P},\theta_{2}} & \ldots & \sigma_{\theta_{P}}^{2} & 0 & 0 & \cdots & 0\\ 0 & 0 & 0 & 0 & \sigma_{\gamma_{1}}^{2} & 0 & \cdots & 0\\ 0 & 0 & 0 & 0 & 0 & \sigma_{\gamma_{2}}^{2} & \cdots & 0\\ \vdots & \vdots & \vdots & \vdots & \vdots & \vdots & \ddots & \vdots \\ 0 & 0 & 0 & 0 & 0 & 0 & 0 & \sigma_{\gamma_{v}}^{2} \end{array}\right) \end{array}$$

The information matrices are expanded as the test moves in a similar way to ensure that all *v* testlets that had been answered so far are represented. The information matrix **I**_*v*_(**θ**, **γ**) under the MTIRT model is of size (*P* + *v*) × (*P* + *v*) and contains four submatrices:

(11) $$\begin{array}{l} {\text{I}}_{v}\left(\theta ,\;\gamma \right)=\left(\begin{array}{cc} \text{I }\left(\theta ,\hat{\theta }\right) & \text{I }\left(\left(\theta ,\;\gamma \right),\left(\hat{\theta \;},\hat{\gamma }\right)\right)\\ {\text{I}}^{\prime }\left(\left(\theta ,\;\gamma \right),\left(\hat{\theta \;},\hat{\gamma }\right)\right) & \text{I }\left(\gamma ,\hat{\gamma }\right) \end{array}\right) \end{array}$$

The first submatrix $\text{I }\left(\theta ,\hat{\theta }\right)$ contains entries with regard to the ability dimensions as defined above by Equation (6) and has elements as specified by Equations (7) and (8).

The second submatrix $\text{I }\left(\gamma ,\hat{\gamma }\right)$ is a *v* × *v* diagonal matrix and contains entries with regard to the testlet parameters. The {*m*-th, *n*-th} element of this matrix is given by the second derivative of the log likelihood with respect to **γ**:

(12) $$\begin{array}{l} {\text{I}}_{mn}\left(\gamma ,\hat{\gamma }\right)=-\text{E}\left[\frac{\partial^{2}\ln\;L}{\partial \gamma_{m}\partial \gamma_{n}}\right]. \end{array}$$

The explicit form for ${\text{I}}_{mn}\left(\gamma ,\hat{\gamma }\right)$ is given for the diagonal elements by

(13) $$\begin{array}{l} {\text{I}}_{mm}\left(\gamma ,\hat{\gamma }\right)=\overset{z}{\underset{i=1}{\sum }}\frac{a_{mi}^{2}Q_{i}\left(\theta \right)\left(P_{i}\left(\theta \right)-c_{i}\right)\left(c_{i}P_{i}\left(\theta \right)-P_{i}^{2}\left(\theta \right)\right)}{P_{i}^{2}\left(\theta \right){\left(1-c_{i}\right)}^{2}}\\ =\overset{z}{\underset{i=1}{\sum }}\frac{{\left(\frac{\partial P_{i}\left(\theta \right)}{\partial \gamma_{m}}\right)}^{2}}{P_{i}\left(\theta \right)Q_{i}\left(\theta \right)}, \end{array}$$

and for the off-diagonal elements by

(14) $$\begin{array}{l} {\text{I}}_{mn}\left(\gamma ,\hat{\gamma }\right)=\overset{z}{\underset{i=1}{\sum }}\frac{\frac{\partial P_{i}\left(\theta \right)}{\partial \gamma_{m}}\times \frac{\partial P_{i}\left(\theta \right)}{\partial \gamma_{n}}}{P_{i}\left(\theta \right)Q_{i}\left(\theta \right)}=0, \end{array}$$

where *z* is the number of items that had been presented for a testlet.

The third submatrix ${\text{I}}_{mr}\left(\left(\theta ,\;\gamma \right),\left(\hat{\theta \;},\hat{\gamma }\right)\right)$ contains entries with regard to both, the ability dimensions and the testlet parameters:

(15) $$\begin{array}{l} {\text{I}}_{mr}\left(\left(\theta ,\;\gamma \right),\left(\hat{\theta \;},\hat{\gamma }\right)\right)=-\text{E}\left[\frac{\partial^{2}\ln\;L}{\partial \gamma_{m}\partial \theta_{r}}\right]. \end{array}$$

The explicit form is given by

(16) $$\begin{array}{l} {\text{I}}_{mr}\left(\left(\theta ,\;\gamma \right),\left(\hat{\theta \;},\hat{\gamma }\right)\right)=\overset{z}{\underset{i=1}{\sum }}\frac{a_{mi}a_{ri}Q_{i}\left(\theta \right)\left(P_{i}\left(\theta \right)-c_{i}\right)\left(c_{i}P_{i}\left(\theta \right)-P_{i}^{2}\left(\theta \right)\right)}{P_{i}^{2}\left(\theta \right){\left(1-c_{i}\right)}^{2}}\\ =\overset{z}{\underset{i=1}{\sum }}\frac{\frac{\partial P_{i}\left(\theta \right)}{\partial \gamma_{m}}\times \frac{\partial P_{i}\left(\theta \right)}{\partial \theta_{r}}}{P_{i}\left(\theta \right)Q_{i}\left(\theta \right)}. \end{array}$$

The forth submatrix is the transposed of Equation (15).

In the course of testlet-based MAT, after the completion of each testlet, the testlet information of each candidate testlet d^*^ is calculated conditional upon the provisional ability vector $\hat{\theta }$ based on Equations (9), (10), information matrices **I**_*v*_(**θ**, **γ**) as specified by Equation (11), ${\text{I}}_{v}\left(\theta ,{\text{u}}_{d^{*}}\right)$, and **Φ**_*v*_ as given in Equation (10). ${\text{I}}_{v}\left(\theta ,{\text{u}}_{d^{*}}\right)$ is the information matrix for **θ** of the responses ${\text{u}}_{d^{*}}$ to the items of candidate testlet d^*^. It has the same form as specified by Equations (11) to (16), with the difference that it represents only one candidate item d^*^ and is not summed across the *t or z* presented items. Analogous to Equation (5), the item selection criterion under the MTIRT model is:

(17) $$\begin{array}{l} |{\text{W}}_{v+d^{*}}|=|\Phi_{v}^{-1}+{\text{I}}_{v}\left(\theta ,\;\gamma \right)+{\text{I}}_{v}\left(\theta ,{\text{u}}_{d^{*}}\right)|. \end{array}$$

The testlet with the largest determinant of the matrix ${\text{W}}_{v+d^{*}}$ is selected to be presented next to the participant.

## Research questions

The multidimensional TIRT model we defined in Equation (3) is a relatively complex model because the testlet effects γ_*jd*(*i*)_ are person- and testlet-specific. In practice, besides the ability dimensions, an additional dimension needs to be estimated for every testlet. The combination of the fact that parameters have to be estimated from a very limited number of data points with the high dimensionality makes the estimation of testlet effects a challenge in itself (see Jiao et al., 2013 for estimation of the one-dimensional TIRT model). Thus, the first research question focuses on the question of whether testlet effects can be recovered for typical item pools using the new model. Since the testlet effects are specified as systematic variation of the response behavior caused by the grouping of items to testlets not affecting the mean of the ability estimates, the research question focuses on the variance of the testlet effect:

*Research Question 1*: Can an unbiased estimation of the testlet effect variance be established with the newly formulated MTIRT model for item sets mimicking operational item pools?

The results regarding research question 1 will provide an insight into the breadth of the applicability of the MTIRT model. Additionally, an unbiased estimation will provide a sound justification for the detailed analyses carried out to answer the following research questions, which focus on specific aspects of the proposed method. In order to keep the study design manageable, more general aspects are not covered by our research questions if they are not specific to MAT-MTIRT or if their impact on the performance of the new method can be derived from previous research. Nonetheless, some of these aspects, such as the effects of the relationship between the measured dimensions and the model complexity, are relevant from a practical point of view and are thus picked up in the discussion.

From the perspective of possible future applications of the proposed method, possible improvements in the precision of ability estimates that can be obtained with the new method compared to a conventional MAT using a MIRT model (such as the current standard version of MAT) are of upmost interest. To cover a broad range of assessments, such effects should be analyzed with respect to the size of the testlet effect variances and the number of items included in the testlets. Therefore, the second research question focuses on the comparison between MAT with the MTIRT model and MAT with the MIRT model, conditional upon the size of the testlet effect variance:

*Research Question 2:* Which differences in the precision of ability estimates can be observed between MAT with the MTIRT model compared to MAT with the MIRT model in the presence of testlet effects with different variances?

With the third research question, the number of items included in the testlets is addressed:

*Research Question 3:* Which difference in the precision of ability estimates can be observed between MAT with the MTIRT model compared to MAT with the MIRT model for testlets including different numbers of items?

A realistic variation of (a) the size of the testlet effect variances and (b) the number of items in a testlet allows the results to be generalized to a broad range of operational item pools.

Lastly, it may be possible that a randomly and thus non-adaptively selected set of testlets (RAN) used in combination with the MTIRT model will already lead to a satisfying level of measurement precision of the ability estimates and that moving to MAT will not add a significant increment in precision. If this is the case, moving from MIRT to MTIRT would be sufficient and the effort of implementing a MAT system might not pay off. Thus, the additional potential of MAT compared to more traditional non-adaptive testing is focused on in the fourth research question:

*Research Question 4:* Which differences in the precision of ability estimates can be observed between MAT and RAN?

## Methods

The stated research questions were examined with a simulation study. The simulation is based on a full factorial design with the four factors *testlet effect variance* $\sigma_{\gamma }^{2}$ (0.0, 0.5, 1.0, 1.5), *testlet size* (3, 6, 9), *model* (MTIRT, MIRT), and *testing algorithm* (MAT, RAN). The testlet effect variances range from an ideal case of complete local item independence to values that had been reported for testlet based tests (0.5, 1.0), to the value of 1.5 resembling testlets exhibiting high—albeit not unrealistic—levels of local item dependence. With the testlet sizes 3, 6, and 9 items we included two testlet sizes that are frequently used (3, 6), and a very large testlet size as an extreme example that is sometimes used. We used a fully crossed design in order to cover a large range of typical testing situations that are currently in use (i.e., conditions with model = MIRT and testing algorithm = RAN) and to make it possible to examine the effects of: (1) applying the MTIRT model instead of the MIRT model, (Equation 2) selecting items adaptively instead of non-adaptively, and (3) using the combination of MTIRT with MAT for all these testing situations. Thereby, test developers will be able to compare the testing situation which best fits their own circumstances with conditions in which the MTIRT model, MAT, or the combination of both is used.

In every condition, *P* = 3 dimensions were examined. Three dimensions were chosen because this number of dimensions is examined in several operational assessments. Furthermore, it is a good compromise with regard to calculation time between the special case of two dimensions and a larger number of dimensions. However, the method is of course applicable to other numbers of dimensions. For each of the 10 replications and for each cell of the design, *N* = 5, 000 ability parameters were drawn from a multivariate normal distribution with **θ**~MVN(**μ**, **Φ**) where **μ** = (0, 0, 0) and

(18) $$\begin{array}{l} \Phi =\left(\begin{array}{ccc} 1.00 & 0.80 & 0.80\\ 0.80 & 1.00 & 0.80\\ 0.80 & 0.80 & 1.00 \end{array}\right). \end{array}$$

The latent correlations of 0.80 between the three ability dimensions are a careful representation of the height of latent correlations between ability dimensions typically found in large-scale assessments of student achievement (e.g., latent correlations of 0.85–0.89 between the dimensions for student literacy in mathematics, reading, and science in PISA 2012 are even a bit higher; s. OECD, 2012).

Additionally, an item pool was generated that was used in all research conditions. For each of the three dimensions 108 items were generated, each one loading on exactly one dimension. This loading was indicated by setting the *p'*s component of the vector **a**_*i*_ to 1 and the other two components to 0. An item loading on dimension *p* = 1, for example, was assigned the discrimination vector **a**_*i*_ = (1, 0, 0). Hence, between-item multidimensionality was used. It was chosen for the simulation because it is the predominant version of multidimensionality used in operational tests. The item difficulties of the 3 · 108 = 324 items were drawn from a uniform distribution for each of the three ability dimensions, *b*~U(−4, 4). This distribution represents a situation a test developer would strive for when constructing an adaptive test, since enough items are available over a broad ability range (cf. Reckase, 2010). Items were assigned to testlets according to their rank order in item difficulty. The testlet parameters needed for the MTIRT conditions were drawn from a multivariate normal distribution according to the testlet effect of the respective condition, $\gamma_{d}\sim \text{MVN}\left(\mu ,E_{D}*\sigma_{\gamma }^{2}\right)$, with **μ** = (0, 0, 0) and ***E***_*D*_ the *D* × *D* identity matrix.

The generated ability, item, and testlet parameters were used to produce responses based on the MTIRT model from Equation (3). With these responses, the testing procedure was simulated for the different research conditions. In the simulation of the testing procedure, the item difficulties, the item discriminations, and **Φ** were assumed to be known. For the MTIRT conditions, the item difficulties were fixed to the generated values. In the MIRT conditions, the model from Equation (4) with γ_*jd*(*i*)_ = 0 for *d* = 1, …, *D* was used. For this model, it had to be considered that the item difficulties used for response generation are only valid under the data generation model and thus under the MTIRT model in the present case. If the response set based on the MTIRT model were to be scaled with the MIRT model, the resulting item difficulties would correlate nearly perfectly with the original item parameters but would have a smaller variance. This effect is well known and referred to in the literature as “shrinkage” (Wang and Wilson, 2005a,b; DeBoeck, 2008). To account for the shrinkage effect, the MTIRT item difficulty parameters were transformed into MIRT item difficulty parameters. Therefore, the generated data were re-scaled with the MIRT model. Then, a scaling factor was calculated by regressing the item difficulties of the MIRT model on the item difficulties of the MTIRT model^1^. Since the amount of shrinkage depends on the number of items in a testlet and the testlet effect, 12 scaling factors were calculated (see Table 1). Finally, the MIRT item difficulties were obtained by multiplying the item difficulties from the MTIRT model with the condition-specific scaling factor. The resulting item difficulties are correct under the MIRT model.

**Table 1:** **Scaling factors to transform MTIRT item difficulties to MIRT item difficulties by testlet effect variance and testlet size**.

	**Testlet Effect Variance**			
**Testlet Size**	**0.000**	**0.500**	**1.000**	**1.500**
3	1.000	0.925	0.863	0.811
6	1.000	0.927	0.867	0.815
9	1.000	0.930	0.871	0.821

Note that these are the item parameters one would use when adopting the current common practice for applying the MIRT model to a dataset including LID between the items of the same testlet. The item discriminations used in the simulation are not affected by shrinkage or other problems and can be directly used in the MIRT condition. Thereby, by using the rescaled item difficulties and the original item discriminations in the MIRT condition, direct comparability with the results obtained in the MTIRT conditions was established. Furthermore, by using fixed values for the item parameters and **Φ**, the simulation setup represents the typical procedure of large-scale assessments in which these parameters are estimated in a first step and the ability parameters in the second step.

The testing procedure was simulated using SAS 9.3. For the MAT conditions, for both the MIRT and MTIRT model, complete testlets were selected. The first testlet was chosen randomly. Next, the testlet with the maximum summed item information given the provisional ability vector $\hat{\theta }$ was selected based on (5) and (17) for the MIRT and the MTIRT condition, respectively. The estimation of the provisional ability and testlet parameters during the course of the test was achieved by carrying out Bayesian modal estimation using a Newton-Raphson procedure, as described by Segall (1996). For the RAN condition, complete testlets were randomly chosen without replacement from the complete item pool. In all conditions (MAT and RAN), testing was terminated after the presentation of 54 items. Thus, 17% of the items in the pool were presented to each simulee.

At the end of the simulated testing procedure, the MTIRT model included a large number of testlet effects and thus dimensions. The estimation with Newton-Raphson integration used within the course of the test is an appropriate and sufficiently fast method to provide provisional ability and testlet-effect estimates but is not the best method to estimate the final results for this high-dimensional problem. In order to achieve the highest possible accuracy for the parameter estimates which were finally used to answer the research questions, the responses gathered in the simulated testing procedure were therefore scaled using the MCMC method with WinBUGS 1.4.3 (Lunn et al., 2000) fixing the item parameters to the values used for response generation. Thus, the item discrimination parameters **a**_*i*_ were fixed at either 1 or 0 indicating the item loadings on the dimensions, the item difficulties *b*_*i*_ were fixed at the generated values, and the pseudo-guessing parameters *c*_*i*_ were set to 0. For the non-fixed parameters, priors with slightly informative hyperpriors were given by **θ**_*j*_~*MVN* (**μ**_**θ**_, **Φ**_**θ**_) with **μ**_**θ**_ fixed at 0 for each dimension and $\Phi_{\theta }\;\sim W^{-1}\left({\text{V}}_{\Phi },9\right)$ the inverse Wishart distribution with 9 degrees of freedom and variance-covariance matrix ${{\text{V}}_{\Phi }}^{-1}=0$ and $\gamma_{jd\left(i\right)}\sim N\left(0,\sigma_{\gamma_{d\left(i\right)}}^{2}\right)$ with $\sigma_{\gamma_{d\left(i\right)}}^{2}\sim \Gamma^{-1}\left(k,s\right)$ the inverse Gamma-distibution with *k, s* ≈ 0. In order to achieve a good comparability between the conditions, the final scaling in the MIRT conditions was also conducted with MCMC, with the same priors used for given MTIRT but without γ_*jd*(*i*)_. The MCMC method was applied only in this final scaling, because it would have been too slow for the estimation of the provisional ability estimates within the course of the test. The number of burn-in iterations ranged from 14,500 to 80,000 for the final scaling. The burn-in length was determined using the convergence criterion proposed by Geweke (1992) in order to achieve a stable convergence for all examined conditions. The final estimates of one replication were calculated on the basis of the last 500 iterations. To obtain point estimates for the abilities and the testlet variance, expected a posteriori estimates (EAPs) were estimated from the respective posterior distributions.

## Results

In this section, the four research questions of the study are answered. First, results regarding the recovery of the testlet effects with the proposed new multidimensional generalization of the TIRT model are presented. Then, MAT with the MIRT model (MAT-MIRT) is compared to MAT with the MTIRT model (MAT-MTIRT) for different testlet effect variances and different testlet sizes with respect to the precision of the ability estimates. Finally, MAT and RAN are compared.

### Recovery of testlet effect variances

Table 2 shows the average testlet effect variances estimates with the standard errors calculated by the standard deviation across replications. The estimated testlet effect variances are virtually unbiased. Note that, this is also true for the conditions with a true testlet effect variance of 0.000. Thus, using the MTIRT model, which is over-specified in these conditions, does not induce problems.

**Table 2:** **Estimated testlet effect variance by testing algorithm, testlet size, and true testlet effect variance**.

		**True Testlet Effect Variance**							
		**0.000**		**0.500**		**1.000**		**1.500**	
**Testing Algorithm**	**Testlet Size**	***M***	***SE***	***M***	***SE***	***M***	***SE***	***M***	***SE***
MAT	3	0.022	0.012	0.506	0.012	1.012	0.017	1.509	0.024
	6	0.007	0.004	0.505	0.014	0.993	0.027	1.514	0.027
	9	**0.010**	0.005	0.510	0.006	1.015	0.033	1.553	0.045
RAN	3	0.025	0.015	0.495	0.026	1.001	0.020	1.506	0.026
	6	**0.020**	0.008	0.496	0.019	1.012	0.027	1.510	0.032
	9	0.019	0.012	0.511	0.018	1.062	0.043	**1.603**	0.049

*Testlet effect variances whose 95%-credibility interval does not cover the testlet effect variance used for data generation are written in bold face. MAT, multidimensional adaptive testing; RAN, random testlet selection*.

Differences between the testlet effect variances used for data generation and the estimated testlet effect variances are mostly on the second or third decimal. Nevertheless, in some cells of the design, the 95%-credibility interval (±1.96 · *SE*) does not cover the true testlet effect variance. But this is mainly observed in the conditions with a very small standard error in the condition $\sigma_{\gamma }^{2}=0.000$. The third instance where the true testlet effect variance is not covered by the 95%-credibility interval around the estimated testlet effect is observed for a very large testlet effect variance of 1.500, and very large testlets. Taking into account that using a 99%-credibility interval would yield no significant differences and that the magnitude of the differences is small, the estimation shows a very solid pattern. In consequence, all conditions were examined in the further steps of analysis.

### Measurement precision as a function of testlet effect size

The second research question considers the differences in the precision of the ability estimates between MAT-MTIRT and MAT-MIRT with respect to the size of the testlet effect variance. To answer, the average mean square error $MSE=\frac{1}{P}\sum_{p=1}^{P}\left(\frac{1}{N}\sum_{i=1}^{N}{\left({\hat{\theta }}_{ip}-\theta_{ip}\right)}^{2}\right)$ of the three-dimensional ability estimate was calculated. The average *MSE* for MAT-MTIRT and MAT-MIRT for different testlet effect sizes can be obtained from the column titled MAT of Table 3.

**Table 3:** **Average mean square error (***MSE***) of the ability estimates for MAT and RAN under the MTIRT and the MIRT model for different testlet sizes and different testlet effect variances ($\sigma_{\gamma }^{2}$)**.

		**MAT**				**RAN**			
		**MTIRT**		**MIRT**		**MTIRT**		**MIRT**	
**Testlet Size**	**$\sigma_{\gamma }^{2}$**	***MSE***	***SE***	***MSE***	***SE***	***MSE***	***SE***	***MSE***	***SE***
3	0.0	0.150	0.002	0.150	0.002	0.233	0.002	0.234	0.002
	0.5	0.194	0.001	0.196	0.002	0.267	0.005	0.271	0.005
	1.0	0.230	0.002	0.241	0.002	0.296	0.004	0.309	0.003
	1.5	0.261	0.002	0.281	0.003	0.321	0.004	0.345	0.005
6	0.0	0.155	0.002	0.155	0.002	0.238	0.004	0.237	0.004
	0.5	0.229	0.003	0.243	0.003	0.297	0.005	0.317	0.006
	1.0	0.286	0.005	0.329	0.006	0.343	0.004	0.400	0.008
	1.5	0.336	0.005	0.405	0.005	0.386	0.007	0.482	0.007
9	0.0	0.161	0.002	0.161	0.002	0.242	0.003	0.242	0.003
	0.5	0.257	0.003	0.300	0.004	0.326	0.005	0.374	0.007
	1.0	0.329	0.004	0.446	0.005	0.395	0.011	0.516	0.009
	1.5	0.393	0.008	0.581	0.009	0.461	0.016	0.658	0.010

*MAT, multidimensional adaptive testing; RAN, random testlet selection; MTIRT, multidimensional item response theory random effect testlet model; MIRT, multidimensional item response theory model*.

**Figure 2:**
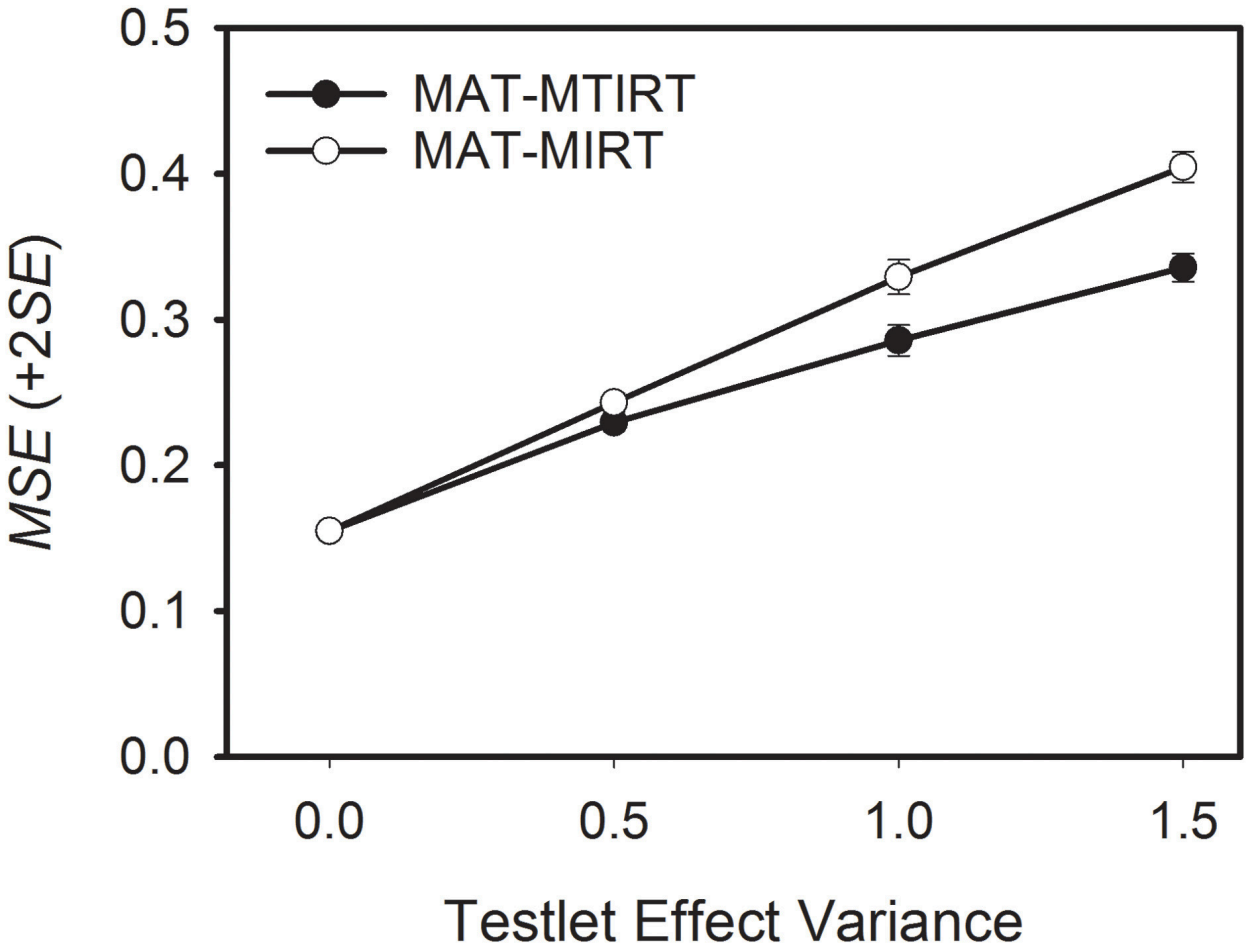
**Average mean square error (***MSE***) of the ability estimates obtained by MAT with the MTIRT and the MIRT model as a function of testlet effect variance**. Testlet size = 6 items.

As a general trend, the measurement precision decreases when the testlet effect variance increases. However, the decrease in measurement precision is smaller if the MTIRT model is used compared to the MIRT model. This can be seen well in Figure 2 which depicts the average *MSE* values for MAT-MTIRT and MAT-MIRT for the case of testlets with six items.

To sum up, two results have to be noted. First, testlet effects lead to a decrease in measurement precision even if the MTIRT model is used. Second, when testlet effects are present (i.e., $\sigma_{\gamma }^{2}>0$), using MAT with the MTIRT model produces more precise ability estimates than the MIRT model.

### Measurement precision as a function of testlet size

**Figure 3:**
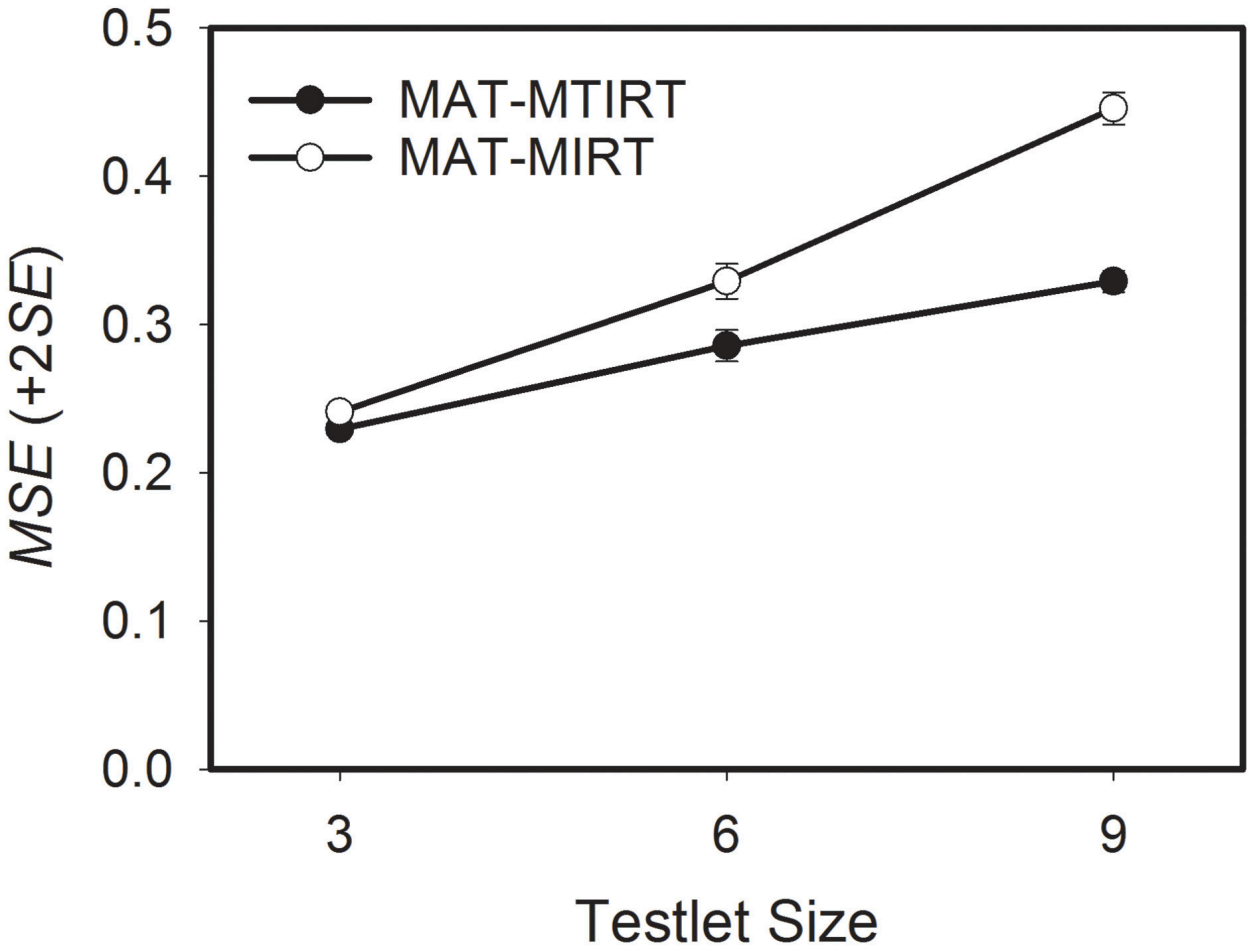
**Average mean square error (***MSE***) of the ability estimates obtained by MAT with the MTIRT and the MIRT model as a function of testlet size**. Testlet effect variance = 1.000.

The third research question focuses on the differences in the precision of the ability estimates between MAT-MTIRT and MAT-MIRT with respect to testlet size. As can be seen in Table 3, the precision of the ability estimates decreases if the number of items embedded in the testlets increases. This is also the case if the testlet effect variance is 0. For MAT-MTIRT and $\sigma_{\gamma }^{2}=0$, for example, the average *MSE* increases from 0.150, through 0.155, to 0.161 for testlets of size 3, 6, and 9, respectively. The differences in *MSE* can be interpreted as the effect of selecting testlets of increasing size which reduces the adaptivity of the testlet selection process. An exemplary graphical representation of the results for a testlet effect variance of 1.000 is shown in Figure 3. It becomes obvious that the effect of the testlet size on the precision of the ability estimates is considerably smaller for MAT-MTIRT than for MAT-MIRT, even though an increase in *MSE* can also be observed for MAT-MTIRT.

To summarize, increasing the size of testlets leads to a loss in measurement precision in MAT. This loss is smaller for the MTIRT model than for the MIRT model if testlet effects are present.

### Differences between MAT and RAN

**Figure 4:**
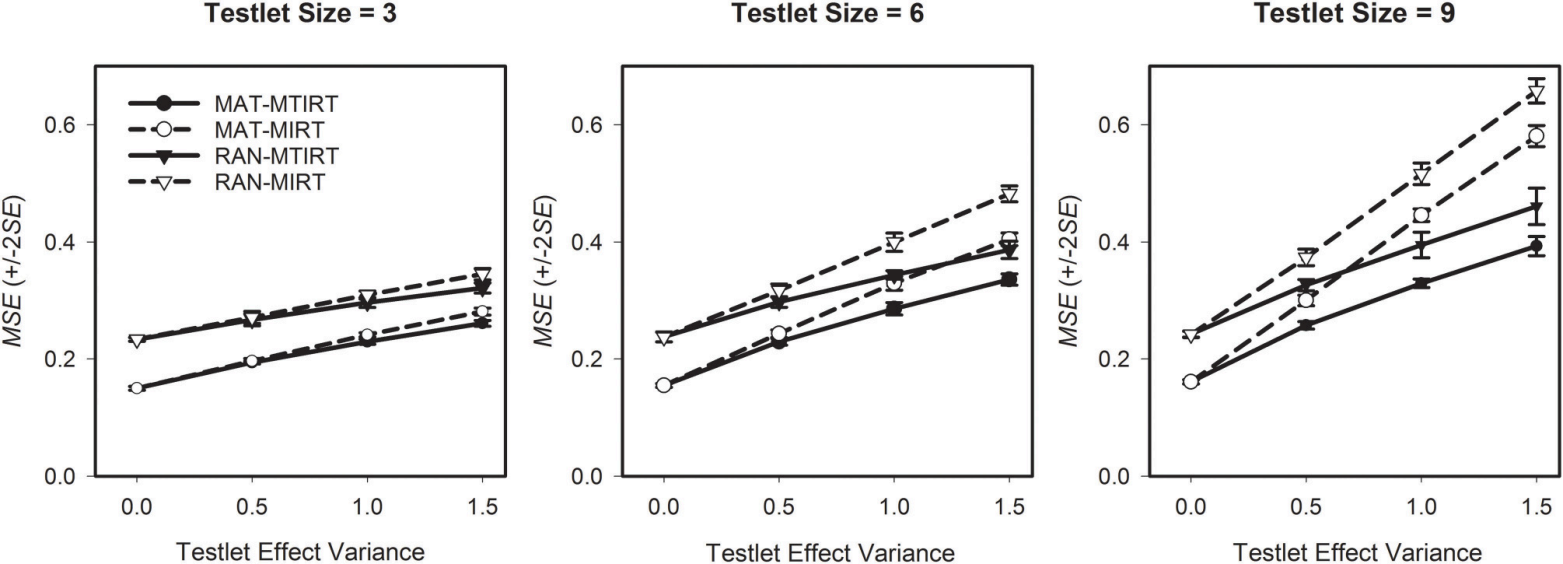
**Average mean square error (***MSE***) of the ability estimates for four combinations of testing algorithm and measurement model by testlet effect variance for testlet sizes three, six, and nine**.

Research question four asks which differences in the precision of the ability estimates can be observed between MAT and RAN. The results in Table 3 provide a differentiated insight into the interaction between testing algorithm, measurement model, testlet effect variance, and testlet size. The first thing to be noted is that for testlets of a comparably small size of three items, the *MSE* is generally lower for MAT than for RAN (Figure 4, left pane). Thus, in these cases, MAT achieved a higher precision of the ability estimates than RAN. When testlet effects are present (i.e., $\sigma_{\gamma }^{2}>0$), the highest precision is observed if MAT is used in conjunction with the MTIRT model. If the response data does not include a testlet effect (i.e., $\sigma_{\gamma }^{2}=0$), there is no difference between the measurement precision of the MIRT and the MTIRT model.

However, with increasing testlet size, the flexibility of MAT is more and more restricted. Correspondingly, the relative importance of the measurement model (MTIRT vs. MIRT) compared to the testing algorithm (MAT vs. RAN) gets larger with increasing testlet size. For example, for very large testlet effect variances of $\sigma_{\gamma }^{2}=1.5$, RAN in combination with the MTIRT model outperforms MAT-MIRT (i.e., has smaller *MSE*) in terms of measurement precision (Figure 4, middle pane). This effect is even more pronounced for very large testlets of nine items each (Figure 4, right pane). Here, the adaptive algorithm can only select $\frac{54}{9}=6$ testlets before the maximum test length is reached. Accordingly, RAN with the MTIRT model achieved a higher measurement precision than MAT-MIRT for testlet effect variances of 1.0 and above. Thus, even in cases where testlet effects are present, MAT can compensate to a certain degree for applying an underspecified and thus “wrong model” if the size of testlets and the testlet effects are not too strong.

However, the main purpose of the present study was to examine the performance of MAT with the “correct model” for cases where LID caused by testlets exists. Here, the proposed method performed well. If non-zero testlet effect variances were present, the combination of MAT with the MTIRT model achieved the lowest *MSE* and thus the highest measurement precision for all combinations of testlet effect variance and testlet size (Figure 4). The lowest measurement precision of all examined combinations of testing algorithm and measurement model was constantly observed by RAN in combination with the MIRT model; the combination which is currently applied by most large-scale assessments of student achievement.

## Discussion

The present study presents and evaluates a new method, expanding the applicability of MAT to the large group of testlet-based tests. The proposed combination of a multidimensional IRT model incorporating testlet effects with MAT results in an applicable solution capable of overcoming the problem of LID in testlet-based tests. Finding a solution for the issue of LID in testlet-based tests is important because recent research provides strong evidence that LID is present between the items of the same testlet in operational large-scale testing programs. Neglecting this fact leads to overestimated test information, underestimated standard errors and, subsequently, to significance tests which are producing too many significant results. Since many educational assessments are used to make important and sometimes far-ranging decisions, this issue needs to be resolved.

A decisive advantage of the method is that, by utilizing the measurement efficiency of MAT, it makes it possible to apply an appropriate model including testlet effects but without the need to prolong testing sessions. Thus, the proposed method of testlet-based MAT can be used without altering the time frames of large-scale assessments or reducing the amount or grade of differentiation of the measured content.

The suggested combination of the MTIRT model and MAT performed well. First of all, testlet effect variances were recovered satisfactorily. This result is not trivial since the proposed MTIRT model is complex and estimation problems could have occurred. The results further showed that the measurement precision of the ability estimates decreased with increasing amounts of LID and increasing numbers of items within testlets. MAT in combination with the MTIRT model was able to compensate to a certain degree for these decreases but did not fully eliminate them. Hence, losses in measurement precision due to LID within testlets will still have to be assumed even if MAT-MTIRT is used^2^. From a practical point of view, this result also means that it is still important to (a) diminish LID within testlets within the process of item writing and item reviewing and (b) to test for LID between the items of a testlet in the phase of item calibration (e.g., Yen, 1984; DeMars, 2012). Lastly, MAT clearly outperformed RAN in terms of measurement precision for both the MIRT and the MTIRT model. Nevertheless, it is important to note that testlets with relatively homogenous item difficulties were composed for the present study, thus making a high adaptivity of the testlet selection possible. On the other hand, presenting these relatively homogeneous items based on incorrect provisional ability estimation (especially at the beginning of the test) will be disadvantageous compared to using more heterogeneous testlets. Taking both effects together, the results should provide a reasonable picture of a typical testlet-based MAT (where relatively homogeneous testlets would typically be an aim of test construction). Even though the differences between MAT and RAN might be somewhat smaller for already existing operational item pools from non-adaptive tests, the relative differences between the other varied factors (model, testlet effect variance, and testlet size) will not be affected much.

Some more predictions about the performance of the suggested method can be derived from existing research. First, based on simulation results (e.g., Wang and Chen, 2004; Makransky et al., 2013), the relationship between the measured dimensions will have an impact on the precision of the ability estimates. Since the covariances between the measured dimensions stored in **Φ** are used for item selection and ability estimation by the new method, measurement precision will increase when the relationship between the measured dimensions becomes stronger. Second, introducing more item parameters into the model will make it more flexible, which leads to a better (or at least to the same) model fit and a higher measurement precision. Thus, the model fit and measurement precision of the 3PL version of the MTIRT model as shown in Equation (3) will typically be a bit better compared to the 2PL version, whose model fit and measurement precision will in turn be a bit better compared to the Rasch version of the MTIRT model. For empirical data, the difference in model fit between the unidimensional 3PL and the 2PL is often clearly smaller than between the 2PL and the Rasch model (Haberman, 2010). The same can be expected for the MTIRT versions of the models. However, since the selection of an IRT model not only takes the model fit into account, but also the parsimony of the model and other criteria, different large studies have come to different conclusions on which model to use. While, in the United States of America, models with two or three parameters are used relatively often, and for large-scale assessments in Europe and Australia the Rasch model (uni- or multi-dimensional) is predominant, in international large-scale assessments, all three models can be found (OECD studies typically use the Rasch model in combination with the partial credit model, IEA studies typically use the 2PL in combination with the generalized partial credit model). In order to account for these differences, the proposed model was formulated in the most general form as a 3PL version. This version or a restricted version of it can be used, making the approach applicable to the full range of typical IRT-based large-scale assessments.

Note that, the results of the present study regarding measurement precision will only be altered in the sense of a main effect of the correlation between the measured dimensions or the complexity of the IRT model, while the relative differences between the varied factors, model (MTIRT, MIRT), testing algorithm (MAT, RAN), testlet effect variance (0.0, 0.5, 1.0, and 1.5), and testlet size (3, 6, and 9), will not be changed. Both the correlation between the measured dimensions and the model complexity will have no impact on the bias of the ability estimates since asymptotically unbiasedness is a property of the estimator used.

Due to the complexity of the MTIRT model the MCMC method was used for the final estimation. Thus, the proposed combination of MAT with the MTIRT model is limited to assessments where a final scaling of the complete set of responses of a relatively large sample is feasible. In its present form, MAT-MTIRT is hence not suitable for providing instant feedback to individual participants. However, for most large-scale assessments of student achievement like PISA, PIRLS, or TIMSS, it can be applied if testing is carried out using computers. As a possible next step, the item pool and response data from one of these studies may be used to examine the feasibility of MTIRT MAT within a real data simulation study. Thus, in conclusion, we would like to encourage measurement specialists to consider implementing MAT-MTIRT in operational testing programs since it has the capacity to substantially decrease the problems caused by LID between items within testlets.

## Author contributions

AF: Conception of the study, directing the statistical analyses, drafting of the manuscript, approval of the final version to be published, agreeing to be accountable for all aspects of the work in ensuring that questions related to the accuracy or integrity of any part of the work are appropriately investigated and resolved. NS: Substantial contribution to the conception of the study, programming needed for the simulation study (SAS and WinBUGS), conducting the data analyses, reviewing the manuscript critically for important intellectual content, approval of the final version to be published, agreeing to be accountable for all aspects of the work in ensuring that questions related to the accuracy or integrity of any part of the work are appropriately investigated and resolved. SB: Substantial contributions to the interpretation of the study results, technical advice in the planning phase, inspection and correction of technical (mathematical) parts, reviewing the manuscript critically for important intellectual content, approval of the final version to be published, agreeing to be accountable for all aspects of the work in ensuring that questions related to the accuracy or integrity of any part of the work are appropriately investigated and resolved.

## Funding

The preparation of this article was supported by grant FR 2552/2-3 from the German Research Foundation (DFG) in the Priority Programme “Models of Competencies for Assessment of Individual Learning Outcomes and the Evaluation of Educational Processes” (SPP 1293).

### Conflict of interest statement

The authors declare that the research was conducted in the absence of any commercial or financial relationships that could be construed as a potential conflict of interest.

## Footnotes

^1^The re-scaled item difficulties could have also been used in the MIRT conditions. Nevertheless, the item parameters derived from the regression can be expected to be a bit more precise because the regression uses the responses to all items to predict the item difficulties, reducing imprecision for individual items with response patterns randomly deviating from the expected response patterns.

^2^The reported results for the average *MSE* of the ability estimates can be seen as a placeholder for results regarding other deviation-based evaluation criteria, such as the reliability or the standard error of the ability estimates, for which the relative differences between the examined conditions is comparable.
